# Network-on-Chip Irregular Topology Optimization for Real-Time and Non-Real-Time Applications

**DOI:** 10.3390/mi12101196

**Published:** 2021-09-30

**Authors:** Samuel da Silva Oliveira, Bruno Motta de Carvalho, Márcio Eduardo Kreutz

**Affiliations:** Department of Computer Science and Applied Mathematics, Federal University of Rio Grande do Norte, Natal 59078-970, Brazil; bruno@dimap.ufrn.br (B.M.d.C.); kreutz@dimap.ufrn.br (M.E.K.)

**Keywords:** Network-on-Chip, irregular topologies, design space exploration

## Abstract

Network-on-Chip is a good approach to working on intra-chip communication. Networks with irregular topologies may be better suited for specific applications because of their architectural nature. A good design space exploration can help the design of the network to obtain more optimized topologies. This paper proposes a way of optimizing networks with irregular topologies through the use of a genetic algorithm. The network proposed here has heterogeneous routers that aim to optimize the network and support applications with real-time tasks. The goal is to find networks that are optimized for average latency and percentage of real-time packets delivered within the deadline. The results show that we have been able to find networks that can deliver all the real-time packets, obtain acceptable latency values, and shrink the chip area.

## 1. Introduction

With the decrease in the size of transistors, it was possible to put thousands of them into a single silicon wafer, which caused the advent of Systems-on-Chips (SoCs). It was also possible to put several processors communicating together, thus characterizing an MP-SoC (Multiprocessor System-on-Chip). MP-SoCs require more processing in communication, which made the buses obsolete and unable to meet this demand. Because of this, Networks-on-Chips (NoCs) emerged [1,2]. NoCs are scalable and allow reuse, and these qualities make them a more appropriate solution.

NoCs are formatted by a set of routers that are interconnected, thus forming a topology [3,4]. Routers have the function of forwarding the messages from their source to their destination to provide network communication. How routers are interconnected is directly related to network performance [5,6,7], since the routers route the packets to their destinations. Different topology types occupy different areas due to the way the routers are organized.

Generic applications generally use regular topologies because of their easy implementation. However, in some cases, routers can be out of use either due to traffic patterns or because we have more routers than processing elements (PE), consequently generating a larger area and energy consumption than necessary and affecting network performance. When working with specific applications, the use of irregular topologies can bring us better performance in the area, power, and latency since only the necessary routers can be used [3]. At design time, it is possible to explore the possibilities of connection between routers within the topology. This exploration performs a search, varying the number of routers and positions of the source and destination nodes, saving resources, and prioritizing the evaluated metrics.

When working with real-time (RT) applications, the packets must meet the expected deadline, as a delay can lead to failures in the execution of the task. Some techniques tend to ensure that the deadlines of these packets are met. One of them is the use of priorities for RT packets to have a preference at the time of containment. In addition, the use of heterogeneous routers can have a positive impact on the final latency since different types of routers tend to give us various performances, and merging these routers into our topology can help us increase network performance [8].

This work aims to generate irregular networks optimized through design space exploration (DSE), seeking to improve latency compared to a mesh topology. We also seek to increase the percentage of delivered packets within the deadline and decrease the network area by removing routers.

There are works in the literature using only real-time applications [9]. In order to expand on this topic, this paper uses networks with real-time and non-real-time application support. The objective is then to develop an optimization method to explore the architecture to find NoCs optimized for latency values and real-time packets that can meet their deadline. In addition, this seeks to reduce the network area by removing as many routers as possible without abruptly affecting latency and the deadline. Some techniques that aim to improve network performance were used, such as utilizing heterogeneous routers. Other techniques used were the use of real-time packet priorities and preemptive virtual channels on some routers. The network optimization is performed through a genetic algorithm (GA) through the latency and percentage of delivered packets within the deadline.

To simulate the behavior of irregular networks, the IrNoC tool was used [10]. This tool was developed in SystemC TLM [11]. IrNoC is a cycle-accurate simulator with worm-hole switching. The simulator also has two different types of routers, which can be combined to improve the accuracy of the network. The simulation results are passed on to the GA and then back to the tool, seeking better network settings.

This paper is divided into six sections as follows. Section 2 presents some related studies, and Section 3 explains the evaluation metrics used. The proposed irregular topology is described in Section 4, and the experimental methodology and obtained results are presented in Section 5. Finally, in Section 6, we present conclusions and suggestions for future works.

## 2. Related Works

This section will introduce some works on irregular topologies and real-time.

In [12], an irregular network called UTNoC (Undefined Topology Network on Chip) is proposed. Each router can connect to any other topology in this network, and each router can connect to just one processing element. Routing is based on tables, and they are filled through a broadcast stage. In the UTNoC network, each router can have several ports since it can connect with any other router of the topology.

The experiments performed based on the UTNoC network were done in a SystemC simulator. The tool also uses a genetic algorithm that performs the network optimization by removing connections between the routers so that the network performs near the performance of the application graph. The experiments were done based on four different injection rates. The results show that it is possible to obtain an irregular topology with a performance close to the application graph, reducing the number of connections with only a small increase in latency.

The work in [4] proposes the use of irregular topologies for a specific application. The objective of the work is to generate irregular topologies that are fault-tolerant. With the use of the Tabu search algorithm, redundant links are created to have the packet go through if there is a failure in a communication link between two routers.

Topologies were evaluated for latency and fault tolerance. Each solution was generated based on a task graph. Four synthetic applications and four real applications (MPEG and VOPD) were used for the tests.

As a result, efficient and fault-resistant topologies were obtained. It was observed that the network latency estimate decreased as the links increased. The links gradually increased as more flaws were injected. Solutions involving more links than a ring topology were able to achieve both latency estimation and up to 30% random link fault injection.

In [13], a new approach is presented for generating irregular topologies for specific real-time applications. The proposal seeks to form networks that are free of containment and obtain an efficient cost.

The work proposes a complete approach to co-design in embedded real-time systems that are based on Networks-on-Chips. The system starts to be specified from a graph of tasks, and then a co-synthesis is performed and the cost of the system is minimized. The result of the co-synthesis is expressed as an annotated task graph. Finally, the NoC topology is created without contention. Port-based contention is eliminated by proper communication scheduling, and path-based conflicts are removed, assigning non-intersecting routes for potentially conflicting messages. The construction of the NoC topology considers the time constraints of the system, minimizing costs in terms of energy and resource requirements.

As the communication pattern is known as a priori, it is used for schedule computation and transmission and performs routing, taking into account the temporal constraints of the system. The results show that the system’s performance does not deteriorate, and significant resource savings are also achieved.

The work carried out by [14] focuses on the generation of irregular topologies for fault-tolerant NoCs. In order to prevent transient failures, the work in question seeks to generate irregular topologies that are optimized, seeking to meet a demand for real-time applications. A genetic algorithm is used to find irregular topologies optimized for various fault injection scenarios. From an initial topology, links between routers are added.

When the irregular topology is formed, a fault injector randomly distributes up to 15% of faults among the links, making it impossible to carry out communications between the routers. A second genetic algorithm performs the task mapping, trying to minimize the latency delay, so that the deadlines of the real-time applications are fulfilled.

Routing is performed using the shortest path algorithm, looking for the shortest route between two routers. If there is a failure in the shortest path route, this route is not considered, causing the algorithm to calculate another route. The results show that the generated topologies could deliver real-time packets with less than a 1% deadline delay. Average latency was increased by up to 13%.

In [9] the optimization of irregular topologies for real-time applications is performed. In order to decrease the average latency generated by the network and increase the number of packets that meet their deadline, an optimization approach is presented, focusing on hard real-time applications. A heuristic is used to change the mapping of the routers, seeking to find an irregular topology capable of increasing the number of packets that meet the deadline, while, on the other hand, decreasing the average latency of the NoC.

The generated topologies were compared in a binary tree format, which can deliver packets very easily. The results obtained showed that all irregular networks could be more optimized than the tree topology in terms of deadline. The results obtained showed that in terms of deadline, all irregular networks can be more optimized than the tree topology. When addressing the latency metric, half of the generated topologies were able to be further optimized.

Differently from the works shown above, this paper will focus on irregular topologies that address applications containing real-time and non-real-time tasks, in addition to using heterogeneous routers and area reductions. We aim at optimizing the network for values of average latency, packet delivery rate, and area. Table 1 exemplifies the difference between the works presented in this section and the approach used in this paper.

## 3. Evaluation Metrics

This section presents the metrics used to evaluate the proposed network.

### 3.1. Proposed Network Evaluation

The main objective of an NoC is to ensure that all messages are delivered so that the application works well [15]. When working with real-time systems, this concern is more significant, as a delay in communication can compromise the system’s integrity. Hence, the performance evaluation consists of verifying that the network meets the minimum latency and flow requirements.

This paper is intended to evaluate the network for metrics of average latency, the percentage of the RT packets that meet the deadline, and the area reduction, which will be done by reducing the number of routers. The total area of an NoC is built mainly through the number of routers, so we believe that reducing the number of routers expected in the network reduces the area. The percentage of RT packets that meet the deadline is calculated using a counter at each local portal. At the end of the simulation, the number of RT packets sent was compared with those that met the deadline, and the percentage calculation was performed. Latency is usually calculated in clock cycles [16], but in our paper, it is calculated in nanoseconds (ns) since the IrNoC tool works that way. The average latency can then be obtained by the sum of each packet trafficked in the network and divided by the total number of packets. In Equation (1), it can be better observed how this calculation is performed.

(1) $$\mathrm{Average Latency}=\sum_{i=1}^{Nº\;\mathrm{of}\;\mathrm{Pckts}}\frac{\mathrm{Latency}\;\mathrm{of}\;\mathrm{Pcket}}{Nº\;\mathrm{of}\;\mathrm{Pckets}}$$

In this work, heterogeneous routers, preemptive virtual channels, and packet priority techniques improve network performance. The heuristic seeks to optimize the network by merging different routers and changing the mapping of the routers.

### 3.2. Design Space Exploring

For the design space exploring stage, the genetic algorithm was chosen. Although there are more optimized solutions in the literature, GA is easy to implement and can solve the problem with few iterations. The genetic algorithm was implemented using the C++ language (chosen due to its high performance). The function that starts the algorithm generates a first population with random values, which have their genes altered due to the next steps. The objective of the work is the optimization of irregular topologies compared to other topologies, such as mesh. Due to this, the comparison between heuristic methods to solve the problem is unnecessary.

Given a specific application and a set of initial irregular topologies, the GA then seeks to change the network mapping, seeking to generate more optimized networks. To perform this optimization, the GA considers the average latency, the percentage of packets delivered on time, and the number of routers present in the topology.

**Figure 1:**
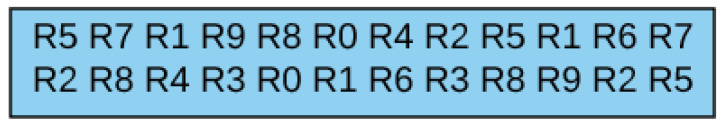
Example of a chromosome in our GA.

A chromosome that is used in our GA represents a solution to the problem. The representation of this chromosome depends on the problem addressed and also on what one wishes to manipulate genetically [17]. In the case of this paper, a chromosome would be the representation of an irregular topology, that is, basically the way the routers are interconnected. The chromosome represents the network mapping, so the chromosome represents how the routers are linked together. In Figure 1, an example of a chromosome used by our GA can be seen. The chromosome has two rows, each row contains the routers present in the network, and the connection between these routers is carried between one router and the one directly below it in the other row.

The initial population is created randomly and starts with a router for each core in the application. Then, the network is evaluated through the fitness function according to established metrics. The GA then removes routers from the topology to optimize in the area. The new mapping generations are tested to find a topology that meets the latency, area, and deadline requirements. The inequality (2) shows the relation as to the number of routers within a topology.

(2) $$\left\lfloor \frac{\mathrm{Cores}\;\mathrm{Number}}{2}\right\rfloor \leq \mathrm{Routers Number}\leq \mathrm{Cores Number}$$

Then, at most, the number of routers will be the same as the cores that the application has and at least the floor of the total number of cores over two. Initially, each router starts with a core associated with it, and as the GA pulls out routers, the cores that will be attached to them will be connected to the routers that remain. In the end, the topology can be up to half of the initial routers since a minor number could compromise the latency and deadline performance of the RT packets. Each router can have a varying number of ports, with four being the limit number, so we can say that each router in our network can connect at most with four other routers. Initially, the network starts with only one type of router, and the GA will test different router types.

**Figure 2:**
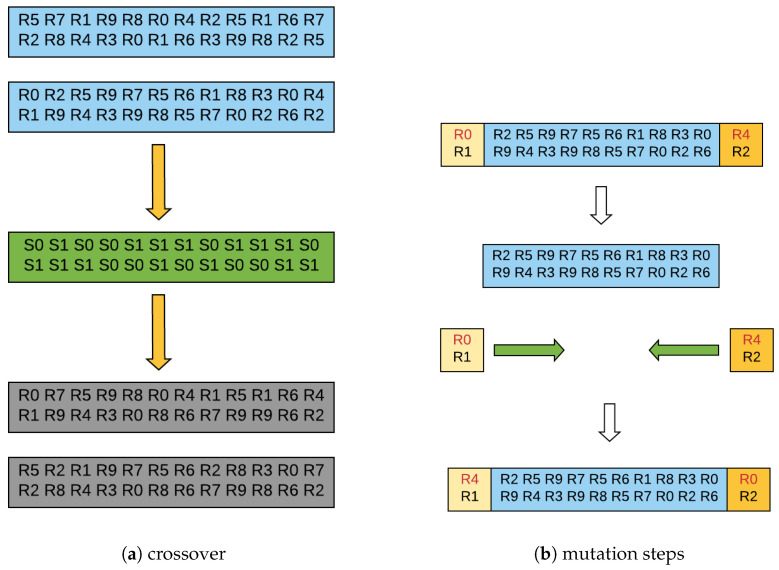
GA operations.

The crossover is accomplished by generating a string of random bits ranging from 0 to 1, and this string is the same size as the parents. Two children are then generated. The first child is made by taking the positions with value 1 in the first line of the bit string and substituting the corresponding number of the exact position of the first parent. The remainder of the row containing 0 values is replaced by the values of the exact position of the first row of the second parent. To fill the second line of the first child, the inversion is done. The values that contain 1 are replaced by the values of the same position of the second row of the second parent, and the remaining values are exchanged for the equivalent of the second row of the first parent. The second child is generated inversely, so there is no risk of repeating connections already tested. In this paper, for the mutation, the gene exchange approach will be used [18]. The first router in the top row is replaced by the last one in the same row in the mutation step. Figure 2 shows this process. The fitness function used is the IrNoC simulator itself. If a population has an invalid individual, the GA tries to generate another valid individual. This process is repeated until the entire population is adequate.

In this paper context, the GA was executed using the parameters in Table 2. The parameters were chosen through several tests with different parameters, seeking to find the most adaptive configuration to the problem. In most experiments, these parameters were evaluated through the convergence points. Using more individuals or a larger population would lead to a much longer simulation time since configurations are tested by changing the number of routers, types of routers, and the interconnection between these routers, leading to an NP-Hard search problem.

## 4. Proposed Irregular Topology

**Figure 3:**
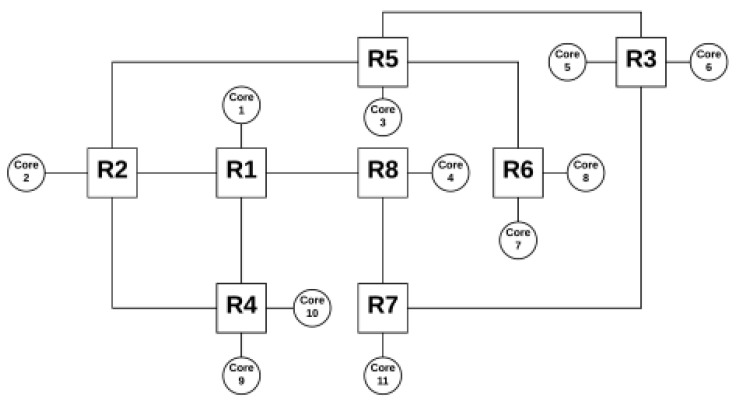
Example of an irregular topology using the proposed architecture.

The architecture proposed here provides more than one core per router, enabling savings in the area. Initially, the architecture starts using a core for each router in the application. As the genetic algorithm executes, it optimizes the architecture by removing routers and allocating the orphaned cores to the remaining routers, leaving a maximum of two cores for each router, thus avoiding a communication overload. When a router has more than one PE connected to it, one of the communication ports between routers is transformed into a local port to accommodate the other PE. Figure 3 exemplifies the architecture used.

The routing algorithm used is the shortest path of Floyd Warshall [19,20] and uses a routing table present in each router, where the routers present in the architecture are presented on each line together with the number of hops to and the outgoing port to reach each router.

The tables in each router are constructed during the formation of the network topology. Each table contains the address of any router present in the topology. Therefore, the higher the topology, the larger the table and its occupied space within the router. As in this paper, the area is measured by the number of routers and not by the space actually occupied by each router and its components, and this is a problem that will not be addressed in this paper but rather in the future. The tables in each router are constructed during the network creation when the routers are each allocated to their position, and they are building and mapping the tables simultaneously.

**Figure 4:**
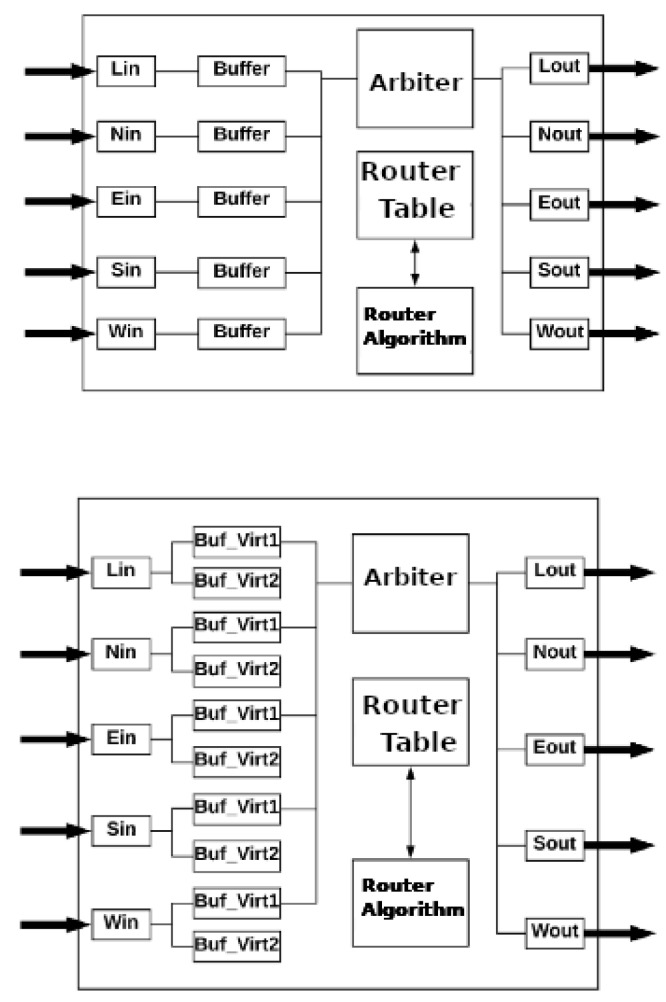
Router models used.

The IrNoC simulation tool has two types of routers, which can be seen in Figure 4. The first type is a simple router, containing only the basic mechanisms of a router. The second type of router differs from the first by adding two preemptive virtual channels for each input port. In the present work, the genetic algorithm merges the different types of routers, seeking to increase the network’s performance. The simulation ends when the RT packet deadline ends since the main goal is to deliver all RT packets within the deadline.

## 5. Experimental Results

This section presents the results obtained from the simulations carried out in networks with irregular topologies and optimized through the genetic algorithm. The simulations were carried out to find networks optimized for average latency and real-time packets delivered within the deadline.

**Figure 5:**
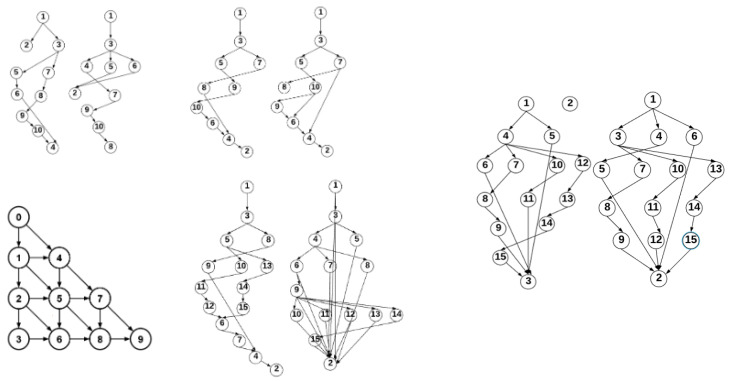
Application communication graphs.

Almost all the applications used are synthetic and generated using the TGFF tool [21,22]. Synthetic applications are those with 15 tasks and the first 4 applications with 10 tasks. The fifth application with 10 tasks is a real application, which corresponds to the Romberg integral method [23], obtained in [24]. Simulations are ended as soon as the RT packets’ deadline ends, after which the percentage of delivered RT packets is checked. Two different sets of applications were generated, with 10 and 15 tasks, respectively. The applications contain different numbers of packets, data volume, deadlines, and traffic patterns. The communication graphs can be seen in Figure 5.

The tests are done for different deadline levels, trying to analyze how far our networks can deliver the packets in real-time. The heuristic seeks to deliver the packets in real-time within the specified period, while for non-real-time packets, delays are allowed. Each application has a different deadline. In the first level, the deadlines established by the simulation tool are used. At the second level, the deadline value for each application drops by half. At the third level, the deadline value is decreased 10 times compared to the first level. The highest deadline value tested for applications is 12,000 nanoseconds because it is an average deadline number for RT applications, and the lowest deadline value used is 500 nanoseconds, which is a small deadline value for an RT application and was chosen to determine if the application would be optimized up to this point.

**Figure 6:**
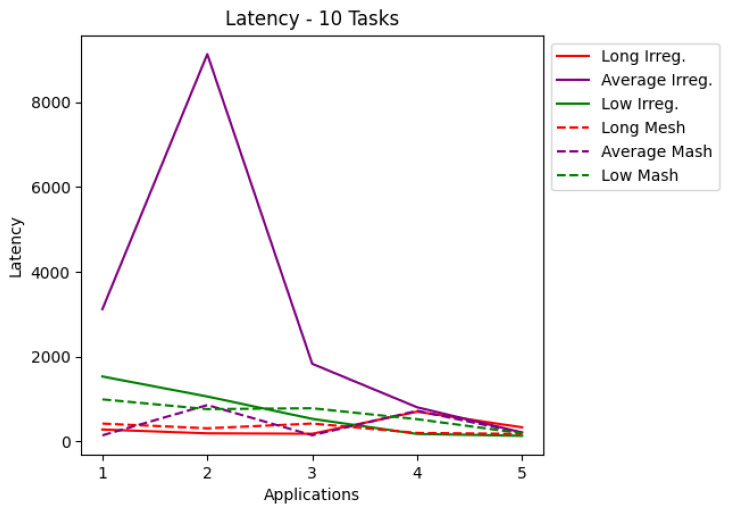
Latency for applications with 10 tasks.

**Figure 7:**
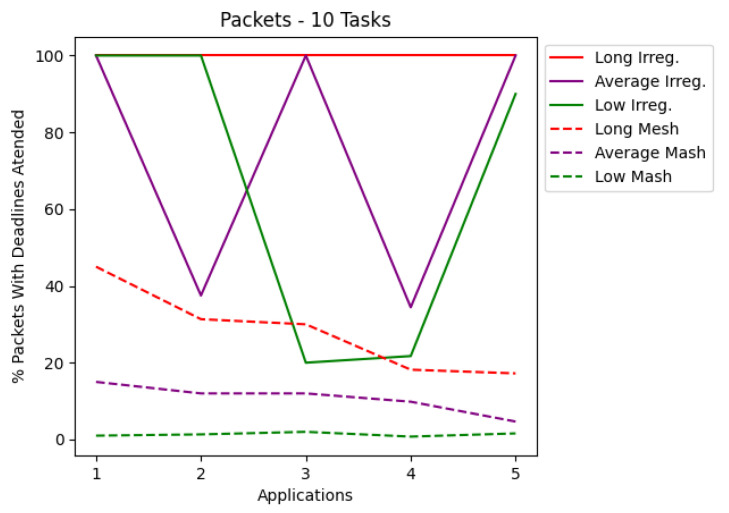
Packets for applications with 10 tasks.

**Figure 8:**
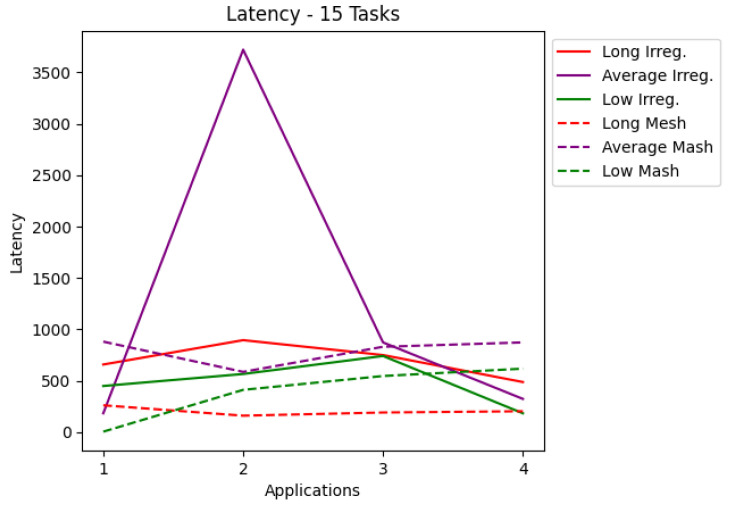
Latency for applications with 15 tasks.

**Figure 9:**
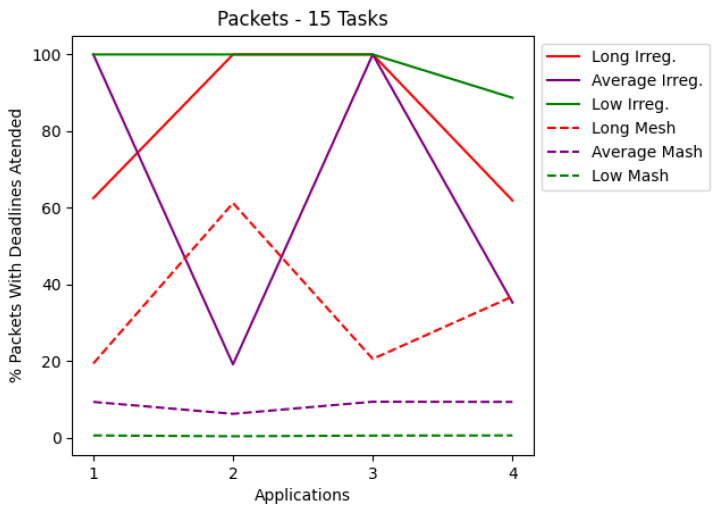
Packets for applications with 15 tasks.

The results are presented in tables and charts. Tables show the application, latency values, the percentage of delivered RT packets, and the number of each type of routers used in the application. T1 is the simplest router, and T2 is the router that contains preemptive virtual channels. The tables present the most optimized result for each application. Each application set was also run on a mesh network that co-handles all application tasks. According to a given number of tasks, the charts cover latency and the percentage of packets complying with their deadlines. Each chart shows the applications for irregular and mesh topologies, differentiating each injection rate used during the simulations. The results are shown in the charts in Figure 6, Figure 7, Figure 8 and Figure 9. The y-axis shows the evaluated metric (latency or packets delivered within the deadline). The x-axis shows the evaluation for each application described in Section 5. Table 3, Table 4, Table 5 and Table 6 present the results in more detail.

### General Analysis

Analyzing the charts shows that for applications with 10 tasks, as shown in Figure 6, the latency in most cases managed to be less than that of the mesh topology. Figure 8 shows that for applications with 15 tasks, the latency tends to be higher than the mesh topology. Figure 7 and Figure 9 show the RT packets delivered within the deadline and show that irregular topologies always managed to deliver more RT packets when compared to mesh topologies. In most cases, 100% of RT packets were delivered on time. All irregular topologies were optimized in terms of area, in some cases reducing by almost half the number of routers initially used. This reduction in the area becomes more significant if we compare it with the number of routers used in the mesh network.

As explained above, in the IrNoC simulator, the simulation process is completed when the deadline ends. Due to this process, the latency in some cases is high, even with few packets delivered. In these cases, the generated topology could not be optimized, causing packets to become congested in the network, avoiding being delivered within the deadline, and overextending latency. When the topology can be optimized properly, it adds the second type of router, which has better support for real-time multi-packet traffic, as it has dedicated channels for this purpose. In addition, by also finding a router interconnection configuration that will favor the application graph, it is possible to manage to deliver all packets within the deadline. This optimization also causes lower latency, including reducing the number of routers, favoring the chip area.

Table 7 shows the values of the T statistical test, using a 95% confidence level. The test used is paired and two-tailed. The sets of applications with irregular and mesh topologies for latency and delivered packets are compared. In the item delivered packets for applications with 10 cores, it was possible to deliver 100% of the packets in most cases. For the tests with a long deadline, all RT packets managed to meet the deadline since they had more time for delivery. In other cases, it was only possible to deliver 100% of the packets in half of the cases. When compared to the mesh topology, it is noted that the optimized irregular topology does better in all cases of delivered packets, also managing in most cases to remove routers from the topology, reducing the area. The T-test shows a large statistical difference for long deadline applications, showing that the approach to a slightly longer deadline is much higher than mesh.

When comparing latency, the *T*-test shows that there was no variation, and in the tables, we can see that the average latency of the mesh topology is lower in some cases. This is because the simulation is stopped as soon as the deadline ends, so in fact, we do not have a total average latency but a partial one until that moment of the packet’s delivery. As the mesh was unable to deliver 100% of the packets at any time, we can assume that in a scenario where the mesh delivers all RT packets, its final average latency would be greater than that of the irregular topology.

For results with 15 cores in the application, the delivery of 100% of the RT packets is also achieved in most cases. In applications with short deadlines, this result is achieved in 75% of the cases, while only half of the applications are successful in other test cases. The T-test shows a significant statistical difference in two of the three cases, which shows the superiority of the optimization technique in the irregular topology.

In the latency issue, the mesh topology once again obtains better results. However, as was highlighted above, these values show a partial result of the latency due to the stop criterion of the simulations. Taking this into account and also that in the T-test, there was a big difference in one of the three cases, we can say that for the latency with 15 cores, the irregular approach also becomes superior. When the network area is observed, it can be seen that in most cases, it is reduced by 46.66%, and when this value is not achieved, a decrease of 33.33% in the area is obtained. When comparing the area with mesh topologies, we achieved a reduction in all applications.

When we compare the results obtained here with [9,12], which are the most similar to our presented approach, we can observe that the results of this paper are satisfactory in optimizing irregular topologies. In [12], only non-real-time applications and one type of router are used, and the routing approach has an additional broadcasting cost when compared to our approach. We have area reduction as an advantage in our approach, besides showing that we can optimize irregular topologies with an urgent demand for packet delivery. In [9] uses real-time packets but does not work with non-real-time packets, as is done in our paper. Merging different types of routers, as we do, allows us to improve latency and packets delivered results when compared to [9]. Due to these comparisons, we can state that the proposed approach managed to find optimized irregular topologies, improving latency and delivering packets within the deadline.

## 6. Conclusions and Future Works

This paper analyzed the use of irregular topologies to obtain networks with optimized values for latency, area, and a higher rate of real-time packets delivered within the stipulated deadline. The networks used are optimized through a genetic algorithm and rely on heterogeneous routers to enhance achieving the desired metrics.

Different applications were used, with different communication patterns and deadlines for the RT packets. The genetic algorithm is responsible for exploring space and design and seeks to find networks that are as optimized as possible regarding medium latency, RT packets delivered, and several routers. The use of heterogeneous routers also contributes to the optimization of the network since one type of router has preemptive virtual channels, which tends to decrease the latency of real-time packets.

The results demonstrate that it is possible to find networks optimized for different applications and packet delivery times. By exploring irregular networks through the genetic algorithm, we could find topologies that met the latency, area, and deadline of the RT packets. Compared with the mesh networks, the irregular networks that use heterogeneous routers were superior and met the design constraints.

As future improvements, we aim at evolving this work using hybrid networks that use wire-bound routers and routers that connect through small antennas, the so-called WiNoCs (Wireless Networks-on-Chips). It is also planned to analyze the energy consumption to verify the efficiency of hybrid networks and heterogeneous routers.

## Figures and Tables

**Table 1:** Related Works Comparison.

Paper	Irregular Topology	Latency	RT Packets	Heterogeneous Routers	Area
[12]	X	X			
[4]	X	X			
[13]	X		X		
[14]	X	X	X		
[9]	X	X	X		
This Paper	X	X	X	X	X

**Table 2:** The tuning of the algorithm in the experiment.

Algorithm	Tuning
GA	Individuals = 50, Iterations = 50, Crossover = 40%, Mutation = 50%, Elitism = True

**Table 3:** Irregular topologies with 10 cores.

Long Deadline					Average Deadline					Short Deadline				
**App.**	**Latency**	**Packets**	**T1**	**T2**	**App.**	**Latency**	**Packets**	**T1**	**T2**	**App.**	**Latency**	**Packets**	**T1**	**T2**
1	279.35	100%	5	1	1	3120	100%	6	2	1	1530	100%	6	2
2	188.10	100%	3	3	2	9140	37.50%	0	10	2	1058	100%	3	3
3	177.90	100%	3	3	3	1830	100%	3	4	3	530	20%	1	9
4	700.15	100%	4	2	4	802.78	34.42%	0	10	4	173.75	21.73%	10	0
5	329.75	100%	0	6	5	209.93	100%	10	0	5	130	90%	4	6

**Table 4:** Mesh topologies with 10 cores.

Long Deadline			Average Deadline			Short Deadline		
**App.**	**Latency**	**Packets**	**App.**	**Latency**	**Packets**	**App.**	**Latency**	**Packets**
1	418.70	45%	1	140.70	15%	1	990	1%
2	306.73	31.33%	2	856	12%	2	760	1.33%
3	419.44	30%	3	142.80	12%	3	780	2%
4	198.63	18.18%	4	720.45	9.84%	4	522.70	0.75%
5	173.16	17.22%	5	203.21	4.67%	5	190.31	1.59%

**Table 5:** Irregular topologies with 15 cores.

Long Deadline					Average Deadline					Short Deadline				
**App.**	**Latency**	**Packets**	**T1**	**T2**	**App.**	**Latency**	**Packets**	**T1**	**T2**	**App.**	**Latency**	**Packets**	**T1**	**T2**
1	658.86	62.51%	0	10	1	184.46	100%	6	2	1	450	100%	6	2
2	895.67	100%	5	3	2	3720	19.14%	0	10	2	566.67	100%	6	2
3	750.99	100%	3	5	3	873.84	100%	2	6	3	741.76	100%	5	3
4	488.39	61.90%	0	10	4	324.33	35.29%	0	10	4	184,46	88.69%	0	10

**Table 6:** Mesh topologies with 15 cores.

Long Deadline			Average Deadline			Short Deadline		
**App.**	**Latency**	**Packets**	**App.**	**Latency**	**Packets**	**App.**	**Latency**	**Packets**
1	262.12	19.37%	1	881.25	9.37%	1	6	0.62%
2	161.37	61.25%	2	587.50	6.25%	2	412.5	0.41%
3	192.47	20.58%	3	831.18	9.41%	3	547.1	0.58%
4	204.56	36.87%	4	873.75	9.37%	4	618.8	0.62%

**Table 7:** *T* test.

*T* Test 10 Cores			*T* Test 15 Cores		
	**Latency**	**Delivered Packets**		**Latency and Delivered Packets**	
Long	0.813	0.001	Long	0.015	0.027
Average	0.173	0.051	Average	0.628	0.076
Short	0.831	0.081	Short	0.662	<0.001
